# Current vector research challenges in the greater Mekong subregion for dengue, Malaria, and Other Vector-Borne Diseases: A report from a multisectoral workshop March 2019

**DOI:** 10.1371/journal.pntd.0008302

**Published:** 2020-07-30

**Authors:** Rebecca C. Christofferson, Daniel M. Parker, Hans J. Overgaard, Jeffrey Hii, Gregor Devine, Bruce A. Wilcox, Vu Sinh Nam, Sazaly Abubakar, Sebastien Boyer, Kobporn Boonnak, Stephen S. Whitehead, Rekol Huy, Leang Rithea, Tho Sochantha, Thomas E. Wellems, Jesus G. Valenzuela, Jessica E. Manning

**Affiliations:** 1 Department of Pathobiological Sciences, School of Veterinary Medicine, Louisiana State University, Baton Rouge, Louisiana, United States of America; 2 University of California, Irvine, California, United States of America; 3 Norwegian University of Life Sciences, Ås, Norway; 4 VectorLink, Phnom Penh, Cambodia; 5 QIMR Berghofer Medical Research Institute, Brisbane, Australia; 6 ASEAN Institute for Health Development, Mahidol University, Nakhon Pathom, Thailand; 7 National Institute of Hygiene and Epidemiology, Hanoi, Vietnam; 8 Tropical Infectious Diseases Research and Education Center, Kuala Lumpur, Malaysia; 9 Institut Pasteur du Cambodge, Phnom Penh, Cambodia; 10 Department of Microbiology and Immunology, Faculty of Tropical Medicine, Mahidol University, Bangkok, Thailand; 11 National Institute of Allergy and Infectious Diseases, Bethesda, Maryland, United States of America; 12 National Center for Parasitology Entomology and Malaria Control, Phnom Penh, Cambodia; 13 US National Institute of Allergy and Infectious Diseases, Phnom Penh, Cambodia; University of Florida, UNITED STATES

## Introduction

**Fig 1:**
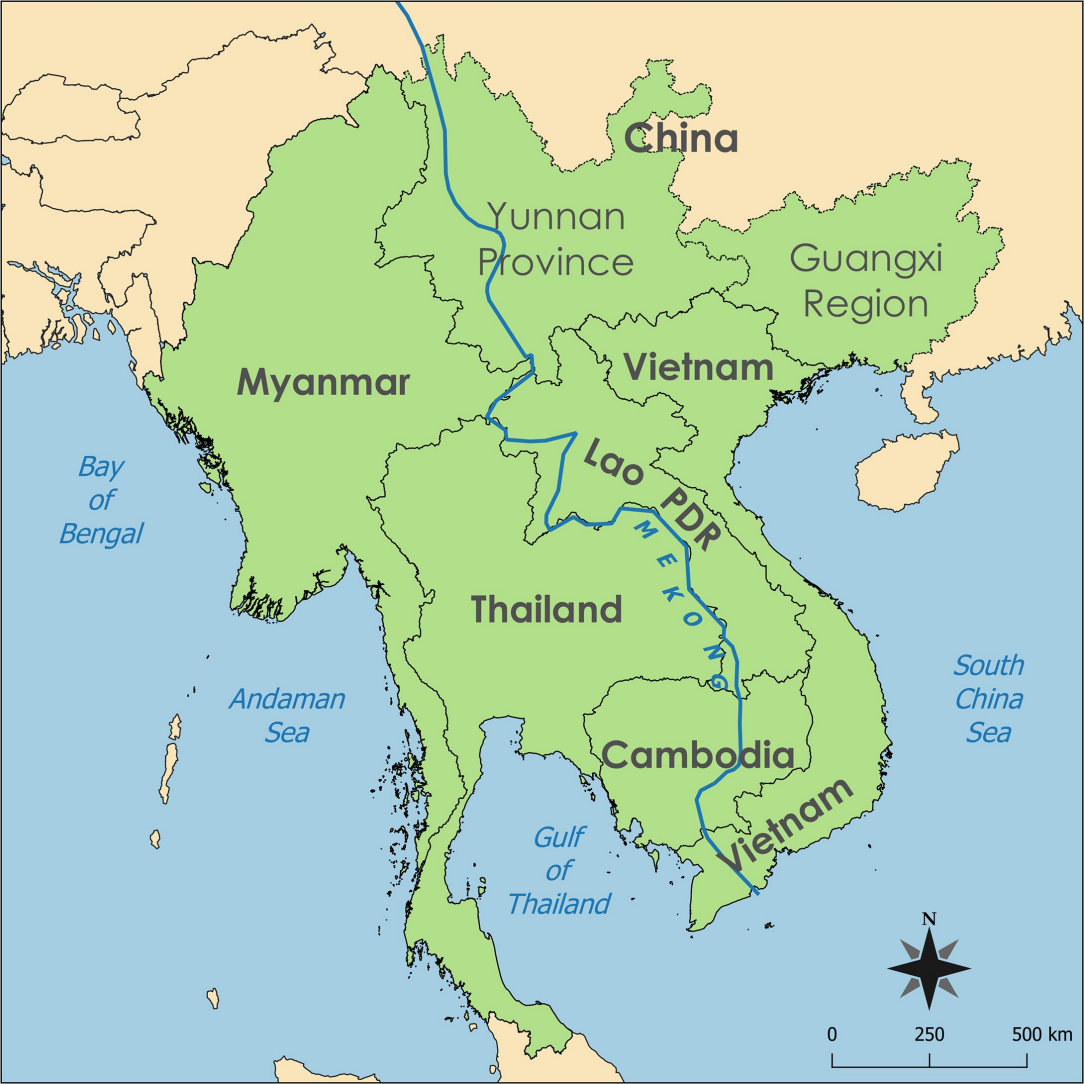
GMS. The map was made using QGIS version 3.4 (https://qgis.org). All map layers were created by one of the coauthors.

Vector-borne diseases (VBDs) are a significant and growing threat to the health of the 326 million people living in the Greater Mekong Subregion (GMS) (**Fig 1**). The GMS is a diverse landscape of cities, rural agricultural communities, forests, deltas, wooded hills, and mountains in the six countries along the Mekong River basin. As the GMS transforms into an increasingly important hub in the global economy, heterogeneous development, rapid urbanization, and socioeconomic risk factors result in increased migration (especially rural-to-urban migration), rapid land-use change, and urban poverty—all factors that can exacerbate transmission of VBDs [1].

Historically, *Plasmodium falciparum* malaria has been a primary focus of public health efforts in the region, and, since the discovery of chloroquine-resistant *P*. *falciparum* malaria in Cambodia in the 1950s, containment of multidrug resistant *P*. *falciparum* malaria has been one of the most pressing public health challenges in the GMS [2]. In response to artemisinin resistance, intense commitments from governmental, nongovernmental, and multilateral agencies have increased access to antimalarials and insecticide-treated bed nets. Additionally, socio-economic development has been associated with dramatic decreases in *P*. *falciparum* cases across the region [3, 4]. As many GMS countries now strive for malaria elimination by 2030 [4], they are simultaneously confronted with other complex health problems, including, but not limited to, (1) arboviral epidemics that are superimposed on decades-long dengue (DENV) transmission and threaten the region’s economy and health [5] and (2) emerging threats such as uncharacterized tick-borne viruses or cutaneous leishmaniasis transmitted by sand flies in rapidly changing landscapes due to land-use change and/or urbanization [6, 7]. Despite a preponderance of research focused on multidrug resistant malaria, DENV, and more recently chikungunya virus (CHIKV) and Zika virus (ZIKV), many challenges remain with regard to the control of these VBDs of public health importance.

In view of these challenges, the United States National Institute of Allergy and Infectious Diseases (NIAID), in conjunction with the Cambodian Ministry of Health, hosted a workshop of 80 experts and government stakeholders in March 2019 from 14 countries (workshop, speakers, and presentation titles are listed in S1 Text) with expertise spanning clinical tropical medicine, ecology, epidemiology, infectious diseases, immunology, vaccinology, vector biology, and virology. The primary goal of this workshop was to prioritize vector research challenges in the GMS in order to better understand transmission and long-term control of VBDs.

Of particular interest was the increasing DENV burden in the region, as it continues to represent a major public health issue for the GMS, particularly in Thailand where cases exceeded 100,000 in 2019 [8, 9]. While cases of DENV continue to rise in the GMS, the overall epidemiology of the virus remains unclear in some GMS countries such as Cambodia where surveillance is limited to clinicosyndromic surveillance in pediatric populations [10]. This constitutes a gap in data necessary to coordinate control efforts within and among the affected countries [11]. Also of interest was the changing landscape of malaria epidemiology, given a 75% drop in case incidence in the GMS since 2018 [4, 12]. With the current goal of elimination by 2030 [4, 13], the overall decline in malaria cases and deaths is attributed to transmission that is increasingly limited to specific geographic locations, strong commitment from policy makers, effective partnerships, crossborder collaborations, and improved access to hard-to-reach group [8]. Understanding the dynamics of malaria transmission across the region will be critical for control programs targeting the remaining geographic and demographic clusters of this disease.

During this March 2019 workshop in Cambodia, it was noted that a group of experts similarly gathered in Singapore in 1977 to discuss the many factors contributing to increases in severe cases of DENV infection (DENV hemorrhagic fever and DENV shock syndrome) in Southeast Asia as well as the current state of knowledge regarding malaria [5]. At that meeting, experts reviewed the recent 1950s malaria elimination campaigns where DDT and chloroquine had failed, and the general consensus was that malaria control would depend upon socioeconomic “progress” in Asia as it had in other locations. Participants correctly anticipated the expansion of both *Aedes* distributions and at-risk, DENV-susceptible populations and discussed approaches that encompassed three major themes: vaccine developments, vector control, and transmission ecoepidemiology.

**Fig 2:**
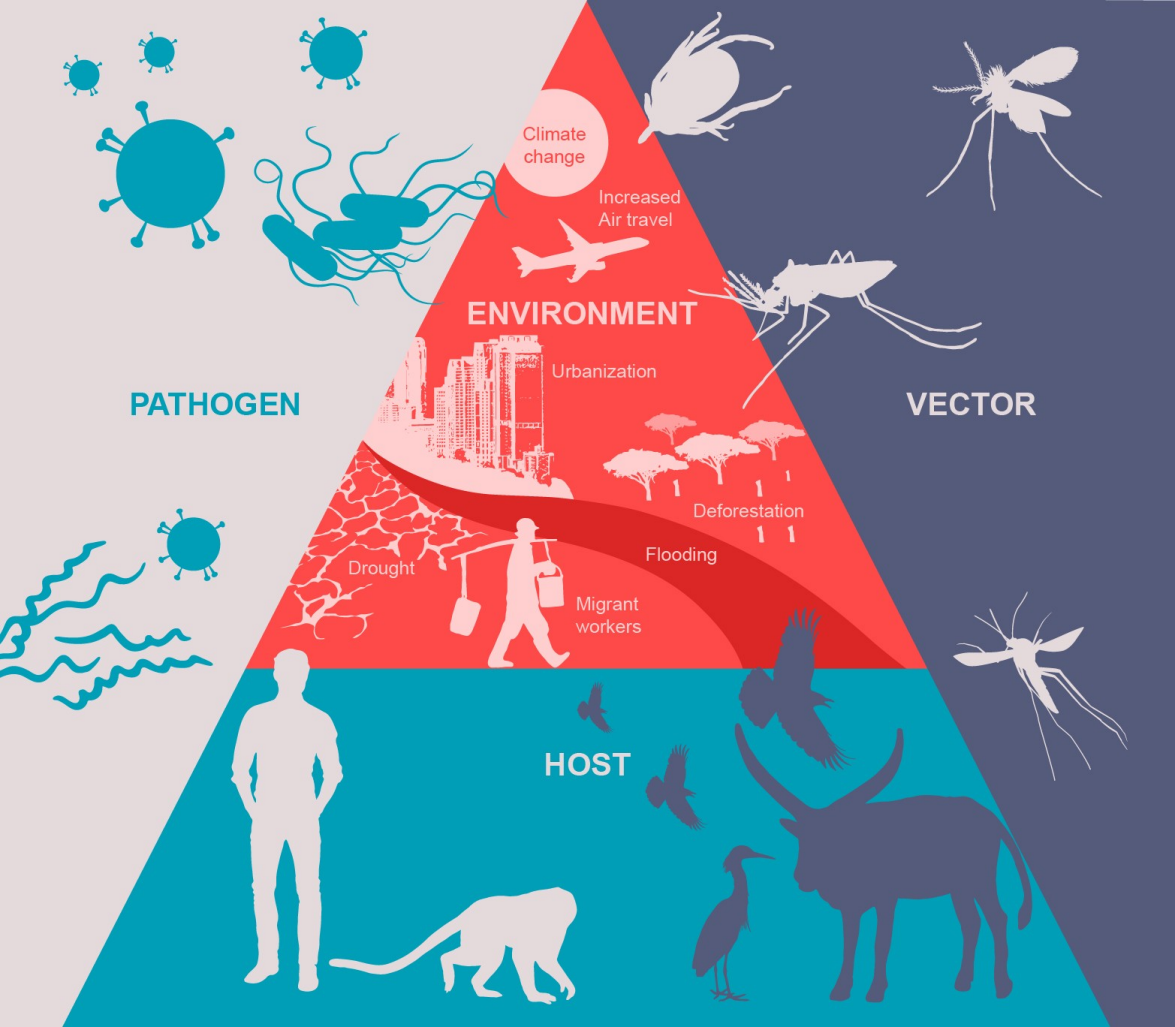
The pathogen–host–vector–environment interface representing the many factors that define transmission.

Discussion of vector-borne disease research often invokes the vector–host–pathogen triad. However, advances in the field have highlighted the importance of considering multifactorial interactions, including the contribution of the environment, which can be defined by ecological, socioeconomic, and/or climatic factors (**Fig 2**). An important goal is to understand how these interactions define transmission patterns in order to identify factors that can be utilized in regionally specific ways appropriate to the GMS yet can still be globally adopted into frameworks where these issues are investigated and translated into actionable items. Thus, in light of progress over the last 42 years, herein we summarize the 2019 workshop presentations, discussions, and newly identified priority outcomes with particular focus on the importance of interdisciplinary basic and translational research of the pathogen–human–vector–environment interface that may lead to the ultimate goal of vector-borne disease reduction, especially for DENV and malaria.

### Vaccine developments

Interventions targeting the pathogen–human interface focus on prevention of infection by vaccination or by antipathogenic medications that can either be used prophylactically or postinfection. Vaccine development for VBDs has been challenging. This is evidenced by the fact that only two vector-borne disease vaccines, the highly effective yellow fever (YFV), and Japanese encephalitis virus (JEV) vaccines, are on the World Health Organization approval list for use without caveat as of 2019 [14]. The recently licensed vaccine for *P*. *falciparum* malaria requires four doses and yields 36% efficacy that wanes with time [15–17]. Efforts at creating an effective and safe DENV vaccine have been frustrated by the cross-reactivity of antibodies among serotypes and unequal immune responses against the different serotypes [18]. In 1977, there was much discussion regarding the potential for monovalent vaccines given simultaneously to produce a tetravalent immune response [5]. At that time, there was still a need to characterize DENV-4 and the unknown genetic diversity of circulating wild-type and future epidemic variants [5, 18, 19].

Prescient 1977 panel members raised concerns around the concept of DENV infection enhancement and serotype-equitable immune responses, which is the required nuance that a DENV-vaccine candidate produce robust yet equivalent levels of neutralizing antibodies to all four serotypes of DENV or else the recipient may have a worsened clinical outcome upon DENV infection. This continues to plague DENV vaccine development even with today’s only currently licensed vaccine [20, 21]. As summarized in S1 Fig from the 2019 presentations, NIAID vaccine development for DENV has taken more than 20 years to carefully address antibody-dependent enhancement (ADE) issues and advance a suitable tetravalent vaccine to Phase III trials [22]. Characterizations of these vaccine candidates—much like those of attenuated viruses presented in 1977—included hundreds of viral constructs. However, current vaccine developers have had the cutting-edge laboratory and clinical tools for high throughput screening of vaccine candidate strains, including derivation of mutants, and improved early-stage clinical evaluation using controlled human infection models [23].

Despite the advances in DENV-vaccine research, critical concerns remain regarding emergence of related mosquito-transmitted flaviviruses, such as ZIKV. The potential for crossreactivity and/or confounding of vaccine immunological indicators highlights the need for tools to elucidate these possibilities [21, 24, 25]. In addition, there is heightened concern about the development of vaccine hesitancy and antivaccination movements leading to a lack of take-up regardless of proven efficacy [26, 27]. Thus, community engagement and education regarding the efficacy and safety of future vaccine candidates will be paramount to the successful uptake of future vaccines for DENV and other VBDs. In 1977, participant experts lauded the novel power of the television for public health educational campaigns to reduce VBDs like DENV and malaria [5]. In 2019, public health agencies must acknowledge the equally powerful role of social media that can both promote, and undermine, vaccine information campaigns [27].

### Novel vector-targeted vaccinology

Advances in vector-based vaccines, such as those targeting mosquito salivary peptides, have also occurred with the progress in pathogen-targeting vaccines. Several studies demonstrate that mosquito saliva coinoculated with the pathogen at the bite site can enhance the likelihood of successful transmission leading to infection as well as the course of arbovirus infection through alteration of the immune responses at the bite site [14, 19, 28–33]. In theory, vaccinated individuals would mount a rapid, Th1-skewed response to pathogens codeposited with mosquito saliva and thus interrupt the pathogen’s ability to establish infection in the dermis [34]. Vector saliva-based vaccines are in the nascent stages of development, with a single Phase I candidate composed of *Anopheles gambiae* salivary peptides [35], but there is growing interest in alternative utilities of vector saliva such as (1) its use as an adjuvant in a pathogen-targeted vaccine as seen in leishmaniasis [36]; (2) its use to differentiate risk of exposure to the pathogen and/or subsequent development of disease [37–46]; or (3) its role in the context of travel medicine. In terms of protection for the traveler, most saliva-specific total immunoglobulin G (IgG) has a duration of less than four months whereby saliva-vaccine induced protection could be appropriately transient, but possibly broad against infections by a vector such as *Aedes aegypti*, which transmits several pathogens of public health relevance (DENV, ZIKV, CHIKV, and YFV) [47].

Transmission reduction is realized when there is a partial or complete interruption of pathogen transfer to the mosquito from the infectious host. There is still a need to understand the within-host dynamics of infection not only with regard to pathogenesis but to better our understanding of transmission. Controlled human infection models, particularly if using vector delivery of pathogens as is done with malaria [48], may support new insights into pathogen dose-dependence of human-to-vector transmission and will help define parameters for effective interventions by vaccines, antimalarials, or vector saliva-based therapeutics for the goals of transmission reduction [49]. In the case of DENV and *P*. *falciparum*, mosquitoes become differentially infected depending on the concentration of pathogen found in the blood meal [50–54]. Symptomatic, asymptomatic, and subclinical disease reservoirs contribute to the transmission cycle [55–57], and laboratory, field, and modeling efforts have shown that the infectiousness of the host can be an important determinant of transmission of several VBD [51–54]. These are important considerations for vector-oriented research in the near future.

### Exposure risk and transmission

Exposure to the vector is the first and necessary process leading to transmission to the human or infection of the vector. Interruption of this contact is also the cornerstone of control, either by directly blocking the contact event (i.e., bed nets) or by decreasing the risk of contact with infectious vectors (through vector population control or reduction of vector longevity). Understanding the processes that drive increases or decreases in human–vector contact are discussed here.

Even with the progress made on vaccines and drug treatments, maintaining long-term control and/or achieving disease elimination will also depend on the interruption of human–vector contact that is the hallmark of vector control programs [58]. Interruption of vector–human contact is primarily achieved via the use of bed nets (insecticide-treated or not); personal protection (clothing, chemical repellents, etc.); and the use of insecticides aimed at the reduction of the vectorial capacity of the local vector populations below the critical threshold needed to maintain transmission [59, 60]. Sprays or fogs that target adult mosquitoes have various efficacy depending on whether it is applied inside or outside the home [61, 62]. Though there is a need for future studies to definitively determine efficacy of outdoor spraying; when supported by real-time patterns of transmission, spatial and temporal analyses can provide evidence of the impact of focally applied measures [63]. Approaches of growing interest include the use of spatial repellents, biological control methods such as *Wolbachia*, and the release of biologically modified arthropods [64].

Insecticide resistance has been and continues to be a major concern in areas that have relied upon the use of insecticides, especially regarding DENV and some malaria vectors in which in many cases there is resistance to multiple commonly used pesticide classes [65–71]. Insecticide resistance in malaria vectors in the GMS is not as well characterized, mainly because it has been difficult to catch or rear sufficient numbers of the primary vectors and perform the necessary resistance bioassays [71–75]. Larviciding for malaria control is a recommended method for larval source management in communities where aquatic anopheline habitats are “few, fixed, and findable” but is not typically used in Southeast Asia for anopheline habitats, although, when utilized, have had success [76–78]. In contrast, larviciding is a key tenet of *A*. *aegypti* control in most urban habitats, although there is widespread resistance to temephos, an organophosphate larvicide commonly used in the GMS, thereby making *Bacillus thuringiensis israelensis* (Bti) or diflubenzuron more promising alternatives [13, 79]. The myriad of accessible and cryptic habitats suitable for *A*. *aegypti* development make larviciding a labor intensive and expensive undertaking that struggles to achieve high levels of coverage. Program efficiency is further reduced by a lack of a consistent, well-trained workforce in the community although there are examples of intersectoral success in Southeast Asia reliant upon nonhealth sectors such as the military, police, education, and construction industry to help control breeding sites [80–84]. Some countries in the GMS rely on community volunteers, especially for larval-based vector control; but often, trained pesticide application workers are hired during peak epidemics and, when the epidemic wanes, so does their workload and compensation. This leads to high turnover and a dearth of trained personnel, causing significant lag time at the height of epidemics. Both community engagement and workforce consistency rooted in the local community are necessary for a continuously efficacious vector control program, and partnering with nonhealth agencies and private industries should be prioritized as governments expand their vector control portfolios [85].

Insecticide resistance is a complex problem, potentially involving a large and population-specific range of physiological, behavioral, and molecular mechanisms. There are no molecular or biochemical diagnostics that can quickly and accurately characterize insecticide-resistant phenotypes or determine the impact of that phenotype on the operational efficacy of insecticides in the field [86–90].

Although peridomestic space spraying, the most commonly applied vector control method during DENV outbreaks, may reduce mosquito numbers for shorter time periods, there is no evidence that it has an effect on DENV transmission [91]. On the other hand, it is peculiar that indoor residual spraying (IRS), a successful malaria vector control intervention, is not more frequently used in DENV vector control. Both IRS, and more specifically targeted IRS, as well as indoor space spraying (ISS) may be effective against both mosquitoes and DENV transmission at high coverage in transmission settings, as they can potentially reduce parasite and pathogen transmission because of the vulnerability of largely endophagic and endophilic mosquitoes [92, 93]. However, such specific methods are only appropriate where there is a high community acceptability and *Aedes* vectors are predominantly endophilic, which may not be the case for *A*. *albopictus* [94]. Although *A*. *albopictus* is considered a secondary vector of DENV, its exophilic nature represents a unique regional challenge for control of other arboviruses such as CHIKV and ZIKV, in addition to DENV [91, 95].

Nonchemical-based alternatives for vector control are also being developed (e.g, biological control [copepods, guppy fish, etc.], genetically modified mosquitoes and improved community engagement strategies). In Vietnam, copepods of the genus *Mesocyclops* were an effective and sustainable biological control agent in the northern and central areas of Vietnam [96], but that has been less consistently adopted in areas of southern Vietnam [97]. Given the context-specific rationales for these biological control tools, current studies of pyriproxyfen and guppy fish in Cambodia suggest the potential for high acceptance and perceived effectiveness of interventions [98, 99]. However, some warn against release of nonnative species such as guppy fish as the overall efficacy of this approach may not outweigh the negative ecological impacts and monitoring for such impacts could be logistically problematic [100]. Community engagement is necessary to carry out any interventional program for vector-borne disease, but Southeast Asia lacks evidence-based community mobilization data, such as that in Central America, but studies are underway in Myanmar and Cambodia ([99, 101], personal communication).

Sterile male techniques aimed at reductions in mosquito populations include irradiation and genetic modifications [102, 103]. Further use of transgenic mosquitoes—both *Aedes* and *Anopheles*—has been put forward as a means of controlling VBDs, although consideration of fitness costs in field settings is critical [104]. However, there are promising strategies, including targeting genes, that result in a reduction of transmission of a pathogen either from the human to the mosquito or vice versa. Additionally, other approaches involve promoting or activating mosquito immune responses against infecting arboviruses that would alter transmission capabilities [105–108]. Biological control methods, such as *Wolbachia* infection of *A*. *aegypti*, are aimed at reducing the competence of vectors and have reduced dengue incidence in the same areas as field trials in Australia and Indonesia, with documented success in arbovirus control in Colombian releases; and controlled release of *Wolbachia*-induced sterile males reduced the *A*. *aegypti* populations in targeted areas in Florida [109–111]. Further, there is interest in the use of endectocides for tackling residual malaria transmission (RMT). This entails treatment of humans or domestic animals with ivermectin in order to kill mosquitoes that feed on either host [112]. This is a suitable approach for the GMS, with its specific problems of RMT, outdoor biting mosquitoes, and zoophagic mosquitoes that are poorly targeted by LLINs [113].

Targeted control methods have been successful in the past and may be successful on very specific spatial scales, as seen in Cairns, Australia [109, 114]. The question of “what happens if the ecological balance is disturbed?” creates two possible deleterious scenarios: (a) further invasions or expansions of secondary vector populations and/or (b) enhanced role of secondary vectors in areas with established transmission. Prevention of reintroduction of vector-borne pathogens is essential, particularly in the populous GMS, and must address the characterization of potential secondary vectors, possible introduction of exotic vectors, or importation of pathogens by visitors and migrants [115, 116].

Additionally, entomological indices to estimate mosquito presence and density rarely correlate with *Aedes*-transmitted arbovirus disease patterns, and thus the primary indicator of successful vector control—a reduction in vector populations—may also not reflect reduced *Aedes*-transmitted arbovirus disease burden patterns [5, 117, 118]. Newer strategies using gravid traps and rapid NS1 testing of mosquitos may be more accurate in assessing disease burden [119, 120]. Methods such as entomological incoculation rates (EIR) and human biting rate (HBR) have had better success with malaria risk assessments in high-transmission settings but are still considered time-consuming, expensive, and imprecise [121–124]. Better methods of determining the nuances of contact rates between infectious mosquito and the affected human population would provide more precision to understand transmission, especially in the context of tracking expanding vector populations. Vector salivary proteins have the potential to become powerful biomarker tools to qualify and quantify mosquito exposure and the risk to mosquito-borne pathogen exposure. Studies have determined that reactivity to mosquito salivary gland extract (SGE) can differentiate mosquito exposure risk due to environmental factors, social factors, and seasonal dynamics [38, 47]. Other studies have shown that serosurveys to SGE and/or particular mosquito salivary peptides can be used to describe differences in vector exposure in space and time due to sociodemographic, environmental, and operational (vector control) factors [37, 39–44]. For example, people living along the Thai-Myanmar border with a high antibody response to *Anopheles* salivary peptide gSG6-P1 had six times higher odds of also being positive to *P*. *falciparum* circumsporozoite antigens and two times higher odds of *P*. *falciparum* infection, compared to low responders to mosquito saliva antigens [45]. For *Aedes* mosquitos, the N-terminus 34 kDa salivary protein was recently used to track the efficacy of vector control interventions in Burkina Faso [125]. The utility of salivary biomarkers may aid targeting *Aedes spp*. vector interventions in places where seroprevalence studies are difficult due to multiple circulating flaviviruses or if disease incidence is underestimated due to the a high proportion of clinically inapparent disease or poor surveillance. Taken together, these data suggest that vector salivary proteins may differentiate mosquito exposure histories in space and time in order to gauge the effectiveness of an intervention and/or the expansion of vector populations (seasonally or permanently).

With regard to other vectors, such as ticks, chiggers, sand flies, and fleas, vector control is not routinely performed in the GMS. Given limited studies and funding available for these vectors and their pathogens, a general consensus concluded that the next step would be better field surveys to characterize the landscape of these vectors and the pathogens that they may carry and transmit. For example, the detection of rickettsial diseases has increased in some areas [126, 127]. Whether these diseases are on the rise or whether this is merely indicative of an increase in surveillance efforts remains to be determined. However, the continued and expanded success of vector control programs and the implementation of novel control strategies requires community buy-in [128]. And while education programs exist across the GMS, consistent compliance is often hard to determine [129].

### Understanding the Eco-Environmental Drivers of Transmission

A common theme across the vector-specific research presentations at the 2019 workshop in Cambodia was a lack of detailed characterization of the ecoepidemiology of VBD systems or how the study of ecological systems of infectious diseases at the population and community levels can inform our understanding of transmission. Environmental conditions, human behavior, and population movement patterns, changes in land use, the microbiome of the vector and habitat, climate change, and intra- and interspecific variability (microbial and vector) all impact observed transmission patterns [130, 131]. The meeting discussion determined that there was a general dearth of understanding of the interrelations of the VBD triad and the further interaction with the environment in the GMS (**Fig 2**) [132–141]. While there were a series of discussion involving mosquito-transmitted infections, tick-borne diseases were also highlighted as emerging health concerns where the epidemiology is tightly intertwined with the ecosystem.

The actual transmission patterns of DENV and malaria, whereas most tick-borne pathogens are in general not understood in the GMS, owing to a lack of disease incidence and prevalence. This is due, in part, to high rates of clinically inapparent infections, where patients do not seek medical services or do not experience any overt illness at all. For example, experts estimate that asymptomatic DENV cases make up one-half to three-quarters of all cases worldwide, while models suggest that these inapparent infections could contribute upwards of 80% to DENV transmission [55, 142–145]. Inapparent infections in other diseases, such as malaria and leishmaniasis, are likely also contributing to a significant proportion of transmission [57, 146–150]. However, patterns of inapparent infection rates are heterogenous, further confounding efforts to estimate transmission intensity [151–155]. While cost-effective and easy-to-use rapid diagnostics exist for DENV, a lack of specific and consistently performing diagnostics contributes to this problem, especially in regions where genetically related viruses cocirculate, leading to misclassification of positive results [156–169]. Diagnostic tools for tick-borne diseases relevant to the region are nonexistent, and this has hampered confirmation of infection, identification of pathogen, and conduct of surveillance. Further, across all VBDs and discussed both in 1977 and 2019, the role of pathogen diversity in transmission intensity, pathogenesis, immune responses/diagnostics, and vaccine development is understudied [133, 156, 169].

An important example of the impact of changing landscapes is RMT. In several areas in Southeast Asia, RMT is more apparent now in forests than in villages [124, 170, 171]. In some study areas, an increase in forest fragmentation (especially encroachment of agricultural fields on forests) led to a reduction in malaria vector diversity and density [140, 141]. In other areas, this pattern has been less clear [172, 173]. In addition to the public health use of chemicals for direct vector control, frequent and widespread use of synthetic pyrethroids and organophosphates in agriculture and livestock industries also contribute to insecticide resistance [174, 175]. Further, land-use change—particularly clearing of primary forests—means that there is an increase in the overall availability of suitable habitats for the establishment and maintenance of *Aedes*-human contact. While more study has been devoted to the impact of rubber plantations on anopheline vectors, the presence of mature plantations can indeed provide increased habitats for *Aedes* vectors [176]. On the other hand, the intensification of agricultural practices (e.g., increased pesticide and fertilizer application, market-driven cropping practices, and areal extension of intensively cropped areas) alters landscapes by reducing the natural forest cover. This landscape change, albeit without urbanization, can still lead to an increase in transmission, as has been observed in the case of DENV and CHIKV in Thailand and Cambodia by providing more suitable breeding habitats for *Aedes spp*. [176–182]. Thus, while urbanization is often the primary land-use change associated with an increase in the risk of exposure to some vectors and their associated pathogens, other changes in land-use (such as agricultural or industrial) should also be considered when assessing risk and subsequently developing plans for mitigation [179, 183, 184].

Changing the environment may also alter the range of not only primary urban vectors, but also those of sylvatic and potential bridge vectors [132, 185]. Human settlement and primary forest clearance patterns; the development of dam, drainage, and agricultural irrigation schemes; and, generally, greater exploitation of natural resources are all drivers influencing patterns of disease distribution and incidence that are often transmission system-specific. All these create new pressures on ecosystems that, in turn, have profound impacts on vector habitats, the carrying capacity of the environment for vector populations, and infectious disease transmission [186, 187].

### Conclusion

Participants at this 2019 meeting recognized a continued need for prioritization into (1) vaccines and therapeutics to prevent and control disease in the human population, (2) understanding the heterogeneity in the exposure risk and factors that define transmission in time and space, and (3) defining the ecoenvironmental drivers that support continued and/or emergent transmission of VBD (Table 1). In many ways, these questions and prioritized vector research echo those raised in 1977 and no doubt in other meetings since (See S2 Fig). However, rather than reflecting a stagnation in science, the 2019 proceedings herein demonstrate that VBD research has evolved to encompass a more in-depth understanding of the factors that govern VBD transmission and control.

**Table 1:** Priority discussion and research questions.

Prioritized Discussions	Priority Research Questions
**Vaccines and novel vector-targeted vaccinology**	How does pathogen diversity contribute to the success of vaccine and/or therapeutic candidates? Is there a role for vector saliva in risk assessments, prophylactics against vector exposure, or diagnostics? How can we leverage continued understanding of the bite-site microenvironmental changes due to vector saliva to aid in development of therapeutics, likely using controlled human infection models? What are the diagnostic needs to accurately surveil and detect outbreaks of VBD in endemic versus epidemic and/or emergent situations?
**Exposure risk and transmission**	What is the contribution of asymptomatic and subclinical infections to the overall transmission? What factors govern the heterogeneity in asymptomatic and/or subclinical rates? How can vector control programs best engage the community as partners for control success as well as to educate for exposure awareness and mitigation? What extrinsic or intrinsic factors govern exposure risk and how do those factors themselves change in the face of anthropogenic pressures? What is the current and future roles of alternative vectors? What is the potential for secondary vectors to adapt and thus increase their public health relevance?
**Eco-environmental drivers of transmission**	How will continued urbanization and land-use changes affect the distribution and relative importance of vector populations? What are the microenvironmental effects of climate change with respect to vector–pathogen interactions that define transmission potential? What is the role of biodiversity in shaping transmission both in endemic and epidemic scenarios? How can basic VBD and vector research be integrated into future development plans to meet the needs of growing populations but still mitigate disease risk?

As our characterizations of specific aspects of these VBD systems have grown more detailed, new questions emerge from these discoveries. This suggests that we have been dealing with a “dangling carrot” model of knowledge. That is, despite ongoing and substantial progress, we have not quite reached our objective of sufficient and necessary understanding of these pathogen–vector–host systems to achieve the desired goal of control, prerequisite for elimination, and, ultimately, eradication of these diseases.

Specifically, 2019 experts felt that pathogen diversity should be considered when developing vaccine, therapeutic, or biocontrol programs to account for the nuances of immune response [188, 189]. In addition, there was agreement that the use of vector saliva has the potential to be used for exposure prophylactic or a determinant of vector exposure risk levels. Given the immunogenicity of vector saliva in the context of pathogen transmission and infection, there was also agreement that development of controlled human infection models could provide important immunological and mechanistic insights into the success of transmission of VBD. Finally, there was consensus that continued surveillance, bolstered by more specific and sensitive diagnosticsm are needed to differentiate etiologies of disease in areas where multiple VBD cocirculate.

Regarding transmission and exposure, the role of asymptomatic and subclinical infections is an important topic as these infectious but undetected individuals are likely reservoirs for continued transmission and subsequent transmission and clinical cases. Education of communities and the engagement in at-risk communities to understand transmission and thus assist in interrupting transmission in culturally sensitive ways is paramount to long-term success of strategies. Additionally, understanding the nuances of spatial and temporality of transmission—including cryptic transmission and zoonotic transmission—is necessary for accurate risk assessment as well as overall realization of successful vector control. Specifically, understanding these nuances would only augment current understanding and study of insecticide resistance and how adjacent vector populations affect the treated vector populations. Finally, the role of alternative vectors, the importation of nonnative competent vectors, and the reestablishment of populations upon the cessation or failure (partial or complete) of current control regimens should be proactively considered.

Finally, continued development through urbanization and varied and changing agricultural practices will likely influence the vector range, vector life-traits, as well as the within-vector kinetics of VBD and should be monitored. Microclimate changes should be taken into account when performing risk assessments, especially as these changes will affect biodiversity of both the vector populations and the pathogen populations. The role of basic biodiversity was thought to be underappreciated in how it shapes the overall transmission landscape, which we know to change in time and space at focal levels, which could lead to fine-scale adaptations that could undermine efforts [64, 190]. The dramatic anthropogenic and environmental changes occurring in the GMS and elsewhere require a paradigm shift with a stronger emphasis on innovative integrated vector management including a synthesis of (1) proven effective methods, (2) new delivery approaches based on proven social theories in the local context, and (3) partnerships with sectors outside of health (e.g., water works, agriculture, forestry, defense, etc.) [191, 192]. The main challenges, however, might be the required change in human behavior and mindsets that such a vector control paradigm requires to be truly effective. How to frame VBD research to incorporate these dimensions arguably is itself a research priority.

Human driven changes, often to meet the needs of growing populations, should be met with basic intersectoral communication whereby VBD biologists and control researchers partner with government, community, urban planners, and other infrastructure experts to meet these needs while mitigating the risk of continued or intensified transmission. Thus, intersectoral collaboration is necessary for future vector research. One widely considered framework is structured around social and ecological determinants of VBD resurgence and the principle modes of environmental change—namely agricultural intensification, urbanization and natural habitat alteration [185, 193, 194]. This theoretical framework emerged from an National Institutes of Health Roadmap to the Future project and emphasizes the complexity of “human-natural systems” in which transmission dynamics are effectively embedded [195]. Understanding such dynamics are critical to further elucidation of the vector–pathogen–host triad, given its complexities spanning the population level (for humans, vectors, and pathogens), community level (human and natural), landscape level, and, ultimately, globally. Cumulatively, the collection of topics raised in this workshop represent current priorities towards achieving the ultimate goals of lessened morbidity and mortality from vector-borne disease in the GMS.

## Supporting information

S1 Text List of presentations and presenters at the GMS Workshop 2019. (DOCX)

S1 Fig Twenty-year journey of NIAID DENV vaccine development. This schematic demonstrates the process and current state of dengue vaccine candidates that have gone from preclinical development through to Phase III trials as of early 2019.
(DOCX)

S2 Fig Observational comparison of topics discussed in 2019 versus 1977. Specific topics (right) were formulated by reading through the notes collected, talk slides, and discussion points raised at the 2019 Phnom Penh meeting and additionally through careful read of the proceedings, papers and discussions of the 1977 Singapore meeting. Materials were again read carefully and the cumulative frequency of mentions (relative size of ribbons) for each discussion point tallied. Ribbon colors correspond to dengue (red), malaria (green), or general VBD (blue) discussion. Ribbon connections to the left panel describe the meeting (1977 and/or 2019) at which topics and disease were discussed. Relative frequency of each topic is proportional to the size of the grey boxes surrounding the topic text.
(DOCX)
