# Supplementary material for: Current vector research challenges in the greater Mekong subregion for dengue, Malaria, and Other Vector-Borne Diseases: A report from a multisectoral workshop March 2019
Source: PLoS Negl Trop Dis. 2020 Jul 30;14(7):e0008302. doi: 10.1371/journal.pntd.0008302 (PMC7392215; doi:10.1371/journal.pntd.0008302)
Supplement: S1 Text — (DOCX) [file pntd.0008302.s001.docx]

**S1 Text**. List of presentations and presenters at the GMS Workshop 2019

| **Speaker** | **Talk Title** | **Affiliation** |
| --- | --- | --- |
| H Auchincloss | Introduction to Defining Vector Research Priorities Workshop on behalf of the US and Cambodia | NIAID |
| H Rekol |  | Ministry of Health, Cambodia |
| F Fouque | Overview of WHO regional surveillance and vector control | WHO Geneva |
| **Session 1: Arboviruses and Current Aedes Vector Research** | | |
| L Rithea | Cambodia National Dengue Control Program | National Center for Parasitology, Entomology, and Malaria Control, Cambodia |
| K Boonnak | Zika and dengue in Thailand | Faculty of Tropical Medicine, Mahidol University, Thailand |
| TV Phong | National dengue control program in Vientam | National Institute of Hygiene and Epidemiology, Vietnam |
| S Marcombe | Insecticide resistance in dengue vectors and alternative strategies in Laos | Institut Pasteur Laos |
| P Ryan | World Mosquito Program: *Wolbachia* in the Mekong | World Mosquito Program, Vietnam |
| S Boyer | Research highlights and future directions of arbovirus vector research at Institut Pasteur Cambodge | Institut Pasteur Cambodge |
| **Session 2: Research Priorities for Anopheles and Considerations for Innovating Vector Control in the Mekong** | | |
| J Hertz | Department of Defense Research Requirements to Mitigate Vector-borne Disease | U.S. Naval Medical Research Unit – 2 Detachment |
| J Hii | APMEN regional priorities overview and next steps | Asia Pacific Malaria Elimination Network |
| T Sochanta | National research on *Anopheles* in Cambodia | National Center for Parasitology, Entomology, and Malaria Control, Cambodia |
| A Vantaux | Malaria transmission in eastern Cambodia | Institut Pasteur Cambodge |
| P Sriwichai | Anophelese vectors and malaria transmission at the Myanmar-Thai border: effective approaches to elimination | Faculty of Tropical Medicine, Mahidol University, Thailand |
| G Devine | Novel insecticidal applications for the Mekong | QIMR Berghofer Medical Research Institute, Australia |
| L Li | Metagenomic pathogen sequencing in mosquitoes | Chan Zuckerberg BioHub |
| G Dimopoulos | Vector-borne disease control using pathogen-resistant transgenic mosquitoes | Johns Hopkins University |
| **Session 3: Unraveling Vector-Host-Pathogen Interactions** | | |
| J Manning | Lab to clinic to field: mosquito saliva-based vaccine update | NIAID Cambodia |
| D Parker | Geospatial co-variation in entomological indices in malaria and dengue systems within the Mekong | University of California Irvine |
| F Oliviera | Intricacies of infectious inoculum in vector-borne diseases: lessons from the sand fly bite | Laboratory of Malaria and Vector Research, NIAID |
| C Mores | Adapting to anthroponotic transmission: what outbreaks can tell us about the future of disease | George Washington University |
| V Munster | Land use change and vector-borne disease | Rocky Mountain Laboratories |
| A Durbin | Dengue controlled human infection models: a tool for vaccine development and more | Johns Hopkins University |
| S Whitehead | Is a dengue vaccine still possible if we can fill the knowledge gaps? | Laboratory of Viral Diseases, NIAID |
| **Session 4 - Other Bugs and New Tools** | | |
| S Davidson | Sandflies and ectoparasites in the Mekong | Armed Forces Research Institute of Medical Sciences, Thailand |
| S AbuBakar | Ticks and tick-borne pathogens: underappreciated emergent health threats | Tropical Infectious Diseases Research and Education Center (TIDREC), Malaysia |
| M Bloom | *Ex vivo* tick organ cultures: gateway to tick-borne virus countermeasure development | Rocky Mountain Laboratories |
| VS Nam | Sandflies: potential vector of leishmania in northern provinces of Vietnam | National Institute of Hygiene and Epidemiology, Vietnam |
| BA Wilcox | Advancing New Tools for Integrated Vector borne Disease Control | ASEAN Institute for Health Development, Mahidol University |
