# Supplementary material for: Current vector research challenges in the greater Mekong subregion for dengue, Malaria, and Other Vector-Borne Diseases: A report from a multisectoral workshop March 2019
Source: PLoS Negl Trop Dis. 2020 Jul 30;14(7):e0008302. doi: 10.1371/journal.pntd.0008302 (PMC7392215; doi:10.1371/journal.pntd.0008302)
Supplement: S1 Fig — This schematic demonstrates the process and current state of dengue vaccine candidates that have gone from preclinical development through to Phase III trials as of early 2019. (DOCX) [file pntd.0008302.s002.docx]

**Figure S1: Twenty-year journey of NIAID DENV vaccine development.** This schematic demonstrates the process and current state of dengue vaccine candidates that have gone from pre-clinical development through to Phase III trials as of early 2019.

**
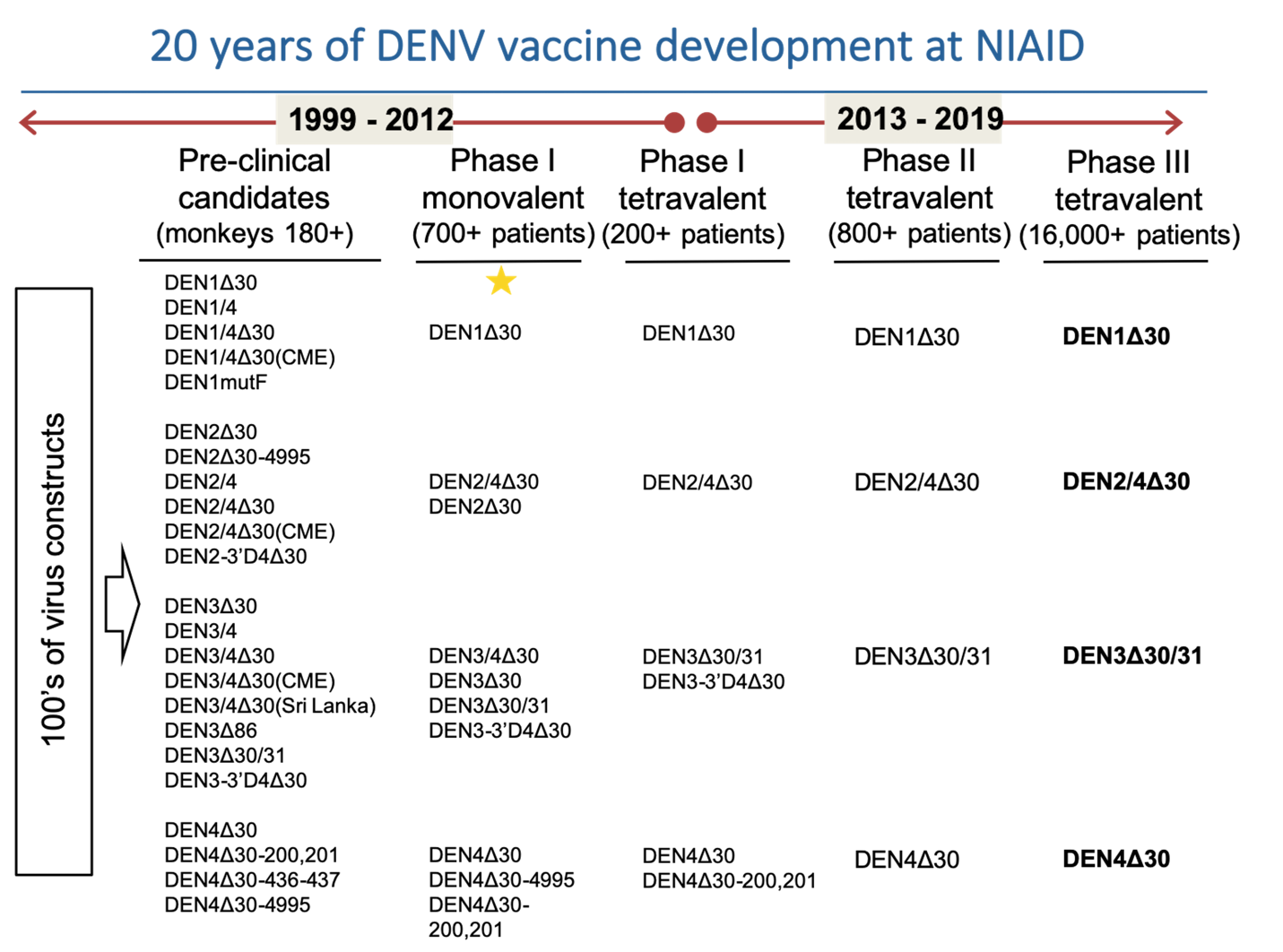
**
