# Supplementary material for: Current vector research challenges in the greater Mekong subregion for dengue, Malaria, and Other Vector-Borne Diseases: A report from a multisectoral workshop March 2019
Source: PLoS Negl Trop Dis. 2020 Jul 30;14(7):e0008302. doi: 10.1371/journal.pntd.0008302 (PMC7392215; doi:10.1371/journal.pntd.0008302)
Supplement: S2 Fig — Specific topics (right) were formulated by reading through the notes collected, talk slides, and discussion points raised at the 2019 Phnom Penh meeting and additionally through careful read of the proceedings, papers and discussions of the 1977 Singapore meeting. Materials were again read carefully and the cumulative frequency of mentions (relative size of ribbons) for each discussion point tallied. Ribbon colors correspond to dengue (red), malaria (green), or general VBD (blue) discussion. Ribbon connections to the left panel describe the meeting (1977 and/or 2019) at which topics and disease were discussed. Relative frequency of each topic is proportional to the size of the grey boxes surrounding the topic text. (DOCX) [file pntd.0008302.s003.docx]

**Figure S2: Observational comparison of topics discussed in 2019 versus 1977**. Specific topics (right) were formulated by reading through the notes collected, talk slides, and discussion points raised at the 2019 Phnom Penh meeting and additionally through careful read of the proceedings, papers and discussions of the 1977 Singapore meeting. Materials were again read carefully and the cumulative frequency of mentions (relative size of ribbons) for each discussion point tallied. Ribbon colors correspond to dengue (red), malaria (green), or general VBD (blue) discussion. Ribbon connections to the left panel describe the meeting (1977 and/or 2019) at which topics and disease were discussed. Relative frequency of each topic is proportional to the size of the grey boxes surrounding the topic text. **
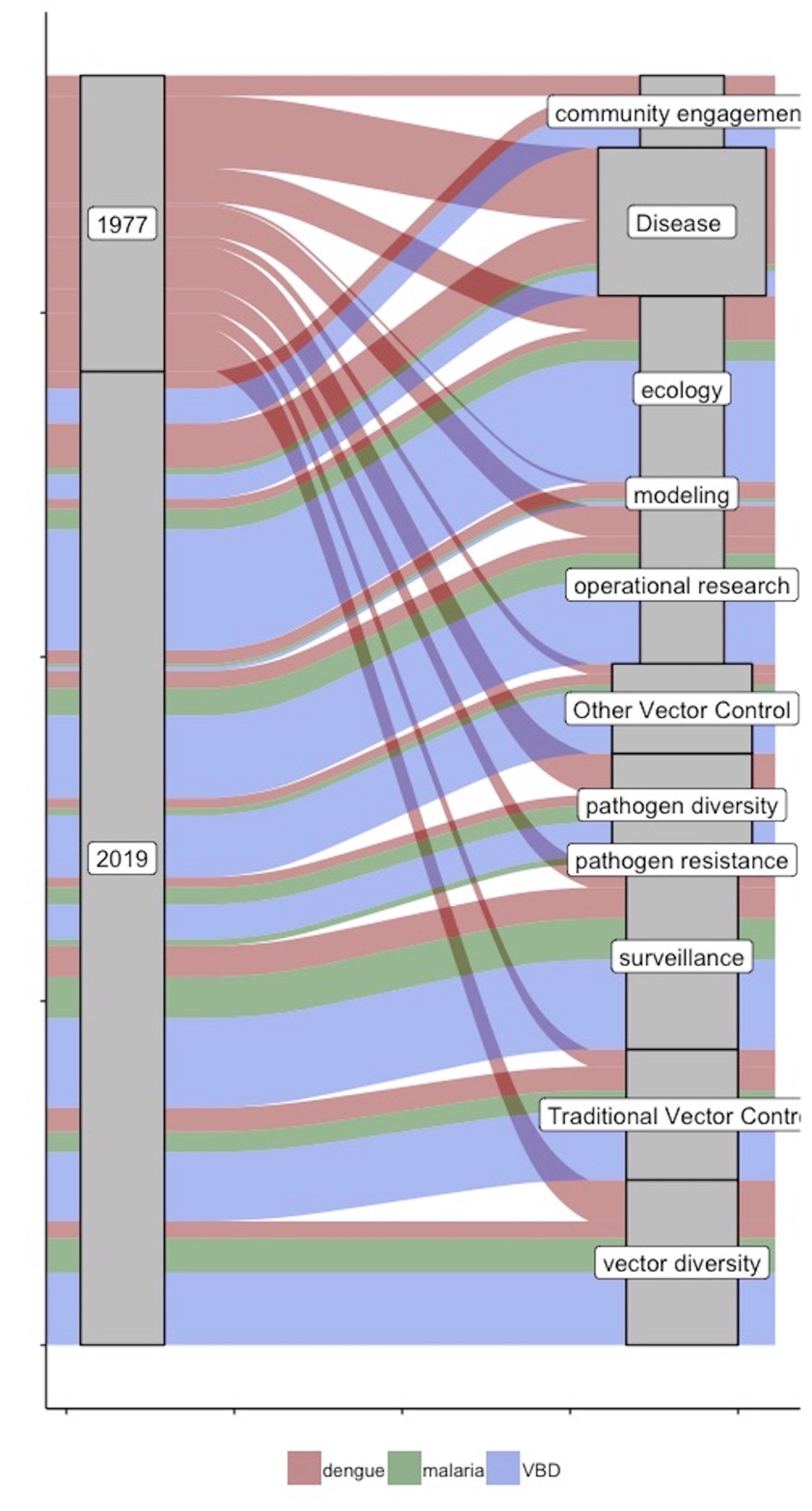
**
